# PCN-224(M)@Fe_3_O_4_/CS/PVA composite nanofibers exhibiting efficient photocatalytic performance for antimicrobial

**DOI:** 10.1016/j.isci.2026.116798

**Published:** 2026-07-16

**Authors:** Chuanmao Huang, Qin Li, Lan Wang, Fenglan Nong, Erjun Sun

**Affiliations:** 1 College of Chemistry, Changchun Normal University, Changchun, Jilin 130032, China

**Keywords:** PCN-224(M), Fe_3_O_4_, electrospinning, antibacterial, photodynamic therapy, photothermal therapy

## Abstract

Bacterial infection caused by drug-resistant bacteria has become a serious social problem and a grave threat to human health. Therefore, the development of antibacterial materials that will not induce bacterial resistance has become a research hot spot. In this paper, a series of nanofibers with high antibacterial performance—PCN-224(M)@Fe_3_O_4_/CS/PVA (M = Zn, Cu, or Fe)—was prepared. These nanofibers combine the photocatalytic antibacterial ability of PCN-224(M), the photodynamic and photothermal effects of Fe_3_O_4_ nanoparticles, as well as the antibacterial activity of CS and the biocompatibility of PVA, exhibiting excellent broad-spectrum antibacterial performance and recyclability. Antibacterial experiments showed that the nanofibers have significant inhibitory effects on Gram-negative bacteria (*E. coli*), Gram-positive bacteria (*S*. *aureus*), and methicillin-resistant *S. aureus* (MRSA). Cytotoxicity test showed that these nanofibers had good biocompatibility and low dark toxicity. This study highlights potential application and value of research on new wound dressings and other biomedical fields.

## Highlights

• Construction of multifunctional antibacterial agents PCN-224(M)@Fe_3_O_4_/CS/PVA
• Excellent antibacterial properties under light illumination
• Synergistic effect from the combination of PCN-224(M), Fe_3_O_4_, and CS
• Electrospun fibers exhibit good biosafety and are easily recyclable

## Graphical abstract

**Figure:**
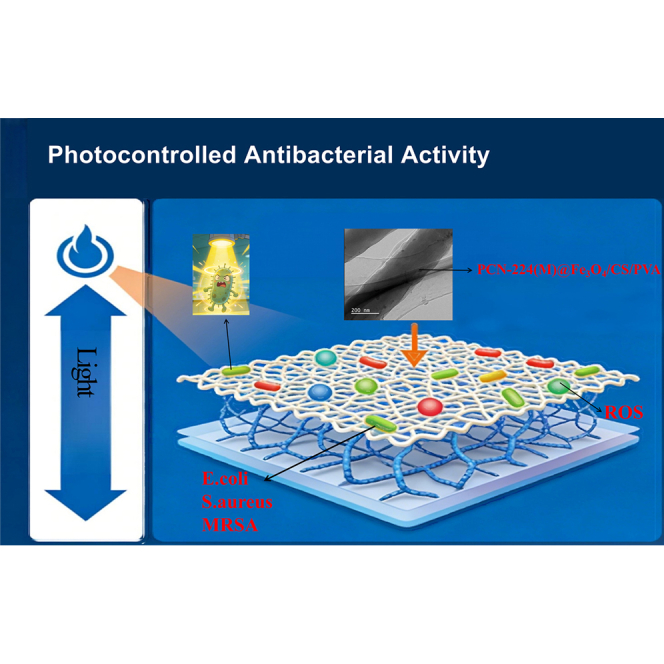

## Teaser

Catalysis; Materials science; Nanomaterials

## Introduction

Diseases, such as endocarditis, tuberculosis, SARS, COVID-19, etc, caused by the spread of pathogenic microorganisms have become one of the most serious threats to the global public health system.^1^^,^^2^^,^^3^ The discovery and use of antibiotics is an essential milestone in the history of human medicine, but their misuse has led to serious threats to human’s life, health, and safety in the form of pathogenic bacterial resistance.^4^^,^^5^ Therefore, there is an urgent need to develop new antibacterial materials to resist bacterial infections in a more effective way without allowing development of drug resistance.

In recent years, various antibacterial therapies based on nanomaterials, such as photodynamic antibacterial therapy (PDAT), photothermal antibacterial therapy (PTAT), and sonodynamic antibacterial therapy (SDAT), have emerged.^6^^,^^7^^,^^8^ PDAT refers to the production of reactive oxygen species (ROS) by photosensitizers through energy transfer or electron transfer under appropriate wavelength light irradiation. ROS can oxidize lipids, proteins, and nucleic acids in microbial bodies, leading to microbial death.^9^^,^^10^ Porphyrin compounds are widely used photosensitizers, but porphyrin molecules are prone to self-quenching in the excited state, thereby reducing their bactericidal effect.^11^ Porphyrin-based metal organic framework (PMOF) are a type of porous material formed by coordination bonds between porphyrin or metalloporphyrin as organic bridging ligands and metal ions/clusters. Porphyrin units are arranged periodically in the structure of MOFs, maintaining a monodisperse state and effectively preventing self-aggregation and self-quenching of porphyrins. At the same time, Metal-organic frameworks (MOFs) have a high porphyrin loading capacity and a porous structure that facilitates the diffusion of singlet oxygen, significantly improving the photodynamic properties of the material.^12^^,^^13^

Porphyrinic zirconium MOFs constructed from zirconia clusters and tetracarboxyphenyl porphyrin (TCPP) organic ligands, such as PCN-222, PCN-223, and PCN-224, have received widespread attention in photocatalytic applications due to their ultra-high specific surface area, large pore volume, and high stability.^14^ However, such materials still face some challenges in practical applications, such as the rapid recombination of photo generated electron hole pairs leading to low quantum efficiency.^15^ The characteristic absorption of porphyrin ligands is mainly limited to the Soret band (∼420 nm) and Q band (550–650 nm), with insufficient utilization of solar spectra.^16^ MOF powder particles are easily dispersed in aqueous solutions and are difficult to recycle.^17^ In order to improve the photocatalytic performance of PMOF materials, researchers have developed various MOF functionalization strategies including metal ion doping,^18^ quantum dot loading,^19^ and heterostructure construction.^20^ Fe_3_O_4_ nanoparticles have high light absorption and thermal conductivity, and can efficiently produce ROS under visible light, exerting strong oxidative killing effect on bacteria.^21^^,^^22^ Combining the photothermal effect of Fe_3_O_4_ nanoparticles with the photodynamic effect of MOF materials can enhance the synergistic effect and improve the photocatalytic ability of the catalyst.^23^^,^^24^^,^^25^ Hariri et al. have developed a novel multifunctional magnetic material, Fe_3_O_4_@SiO_2_@PCN-222(Fe) core-shell microspheres, for efficient dye photodegradation under visible light irradiation.^26^ Designed by Wu et al., a new Fe_3_O_4_@PCN-333 composite was prepared with Fe_3_O_4_ as the core and PCN-333 as the shell, which showed excellent adsorption properties for Tetracyclines (TCs).^27^

Due to the differences in cell membrane structure between Gram-positive and Gram-negative bacteria, the antibacterial mechanisms are also different. Therefore, adopting multi-mode synergistic antibacterial therapy can help achieve broad-spectrum antibacterial activity. Antibacterial activity against *E. coli*, *S*. *aureus*, and MRSA by different porphyrins and PMOFs was studied in different articles (Table 1).^28^^,^^29^^,^^30^^,^^31^^,^^32^^,^^33^^,^^34^

**Table 1:** Recent progress in the antibacterial field in terms of porphyrins and porphyrin-MOFs and comparison

Sample	Antibacterial efficiency against *E coli*	Antibacterial efficiency against *S. aureus*	Antibacterial efficiency against MRSA	Applicable scenarios	Reference
GR@MOF-545	>99%	>99%	N/A	wound dressing, food packaging	Bai et al.^28^
Ag9-AgTPyP	>99.999%	>99.999%	>99.99999%	water treatment, surface antibacterial coating	Cao et al.^29^
pMOF/MnO_2_	not directly tested	not directly tested	>99.999% (5.27 log_10_)	treatment of subcutaneous abscess	Deng et al.^30^
PCN-224(Zr/Ti)	96.4%	N/A	96.2%	medical facility	Chen et al.^31^
UCNP@PCN-224(Pt)/IR808	N/A	N/A	>99.999% (5.27 log_10_)	bactericidal and tumor therapy	Tan et al.^32^
PLLA/Zn-PPy-Ag_2_O	92.6%	91.9%	N/A	neural regeneration	Li et al.^33^
Ag-CuTCPP MOFs	82.18%	89.1%	N/A	drug carriers	Guo et al.^34^
PCN-224(Fe)@Fe_3_O_4_/CS/PVA	99.92% (2.52 log_10_)	>99.99% (2.54 log_10_)	99.96% (2.52 log_10_)	wound dressing, food packaging	This work

Electrospun nanofibers have a crucial application in the field of wound dressings. Nanofibers made from biocompatible polymers can cover wounds such as burns and abrasions, promoting rapid healing without any complications.^35^ Chitosan (CS) is a natural polymer with inherent broad-spectrum antibacterial properties, making it highly suitable as a wound dressing material. However, CS exhibits poor spinnability.^36^ Polyvinyl alcohol (PVA) is a water-soluble polymer that is biodegradable, making it an ideal base polymer for constructing electrospun nanofiber structures. Researchers have already blended CS/PVA and electrospun them into composite fibers for use as wound dressings.^37^ To enhance antibacterial performance, we used natural antibacterial agent CS and PVA with good biocompatibility and high fiber-forming ability as carriers, loaded photocatalyst PCN-224(M) and Fe_3_O_4_ nanoparticles with photothermal effect, and prepared a new multifunctional composite nanofiber material PCN-224(M)@Fe_3_O_4_/CS/PVA through electrospinning technology. The antibacterial activity of the nanofibers was investigated by using Gram-negative bacteria *E coli*, Gram-positive bacteria *S. aureus*, and MRSA as research objects. Research has shown that the synergistic strategy of multiple components can significantly improve the antibacterial performance and biocompatibility of antibacterial materials. At the same time, electrospun fibers are easy to recycle, which can solve the problem of difficult recovery of powdered photocatalysts.

## Results

### Characterization of PCN-224(M)@Fe_3_O_4_ composites

**Figure 1:**
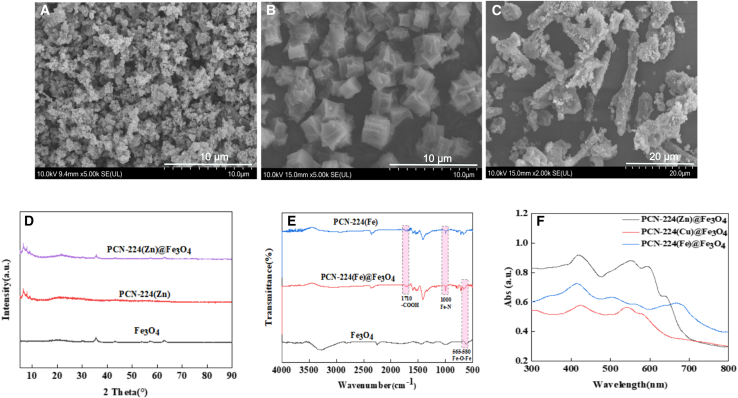
Morphology and characterization of the samples (A) SEM image of Fe_3_O_4_. (B) SEM image of PCN-224(Zn). (C) SEM image of PCN-224(Zn)@Fe_3_O_4._ (D) XRD(d) spectra of Fe_3_O_4_, PCN-224(Zn), and PCN-224(Zn)@Fe_3_O_4_. (E) FTIR spectra of Fe_3_O_4_, PCN-224(Zn), and PCN-224(Zn)@Fe_3_O_4_. (F) UV-Vis DRS spectra of PCN-224(M)@Fe_3_O_4_. Scale bars: 10 μm (A and B) and 20 μm (C).

The morphology and microstructure of pure Fe_3_O_4_, PCN-224(M), and PCN-224(M)@Fe_3_O_4_ composites were characterized by scanning electron microscopy (SEM). As shown in Figures 1A–1C, Fe_3_O_4_ nanoparticles are uniform and spherical, but the agglomeration phenomenon is evident. PCN-224(M) is uniform and massive with a smooth surface. After PCN-224(M) and Fe_3_O_4_ are combined, the surface of PCN-224(M)@Fe_3_O_4_ composite becomes rough, Fe_3_O_4_ nanoparticles are uniformly distributed on the surface of PCN-224(M).

The crystal structures of PCN-224(M), Fe_3_O_4_, and PCN-224(M)@Fe_3_O_4_ composites were examined using powder X-ray diffraction (PXRD). As shown in Figure 1D, the PXRD pattern of the synthesized PCN-224(Zn) corresponded closely to that of the simulated PCN-224(Zn). The characteristic peaks at 6.4°, 7.8°, 9.0°, 11.2° and 13.6° were assigned to the (002), (004), (224), and (006) lattice planes of PCN-224(Zn), respectively.^38^ Fe_3_O_4_ has characteristic peaks at 30.1°, 35.6°, 43.1°, 57.2° and 62.8°, which is consistent with the characteristic diffraction peak of Fe_3_O_4_ in literature.^39^ The X-ray diffraction (XRD) patterns of PCN-224(Zn)@Fe_3_O_4_ composites showed that the diffraction peaks of PCN-224(Zn) and Fe_3_O_4_ coexisted, thereby confirming that Fe_3_O_4_ was successfully deposited onto the surface of PCN-224(Zn). Consistent with expectations, the peak position of PCN-224(Zn) remained unchanged, indicating that PCN-224(Zn) preserved its original framework structure throughout the loading process.

The Fourier transform infrared (FTIR) spectra of Fe_3_O_4_, PCN-224(M), and PCN-224(M)@Fe_3_O_4_ composite materials are shown in Figure 1E. When metal ions are embedded in the TCPP, they coordinate with the nitrogen atoms in the porphyrin ring, thereby promoting the vibrational deformation of the nitrogen ring. Therefore, the presence of M−N stretching vibration characteristic peaks near 1000 cm^−1^ can be observed in both PCN-224(M) and PCN-224(M)@Fe_3_O_4_ composite materials. The absorption peak near 1700 cm^−1^ corresponds to the carboxyl coordination between the porphyrin ligand and the zirconium metal node. In addition, characteristic absorption peaks of 565–580 cm^−1^ can be observed in Fe_3_O_4_ and PCN-224(M)@Fe_3_O_4_ samples, which are caused by the stretching vibration of Fe–O in Fe_3_O_4_.^40^ As shown in Figure 1F, the absorbance properties of PCN-224(M)@Fe_3_O_4_ composites were investigated via UV-vis diffuse reflectance spectra (DRS). In the visible region, the absorption peaks of composites are similar to those of TCPP(M),^41^ but the width of the absorption peak broadens. The consistency of the characteristic peaks between PCN-224(M)@Fe_3_O_4_ composites and TCPP(M) indicates that the composite of Fe_3_O_4_ nanoparticles and the formation of the composite material did not significantly change the inherent π-π∗ electron transition characteristics of the porphyrin ring system. Also, the higher absorption intensity and the red shift of absorption wavelength showed that these composites could absorb visible light, suggesting that they are promising photocatalysts for the photo-driven applications.^42^

**Figure 2:**
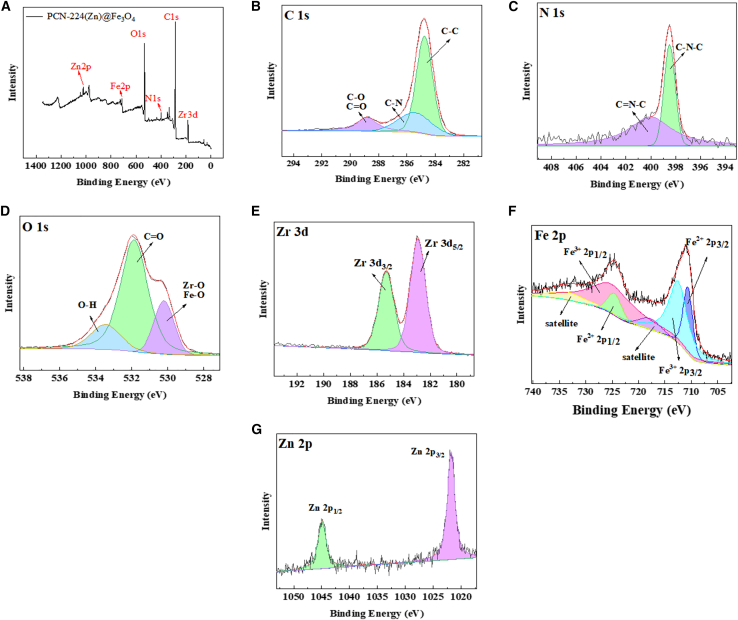
XPS spectra of PCN-224(Zn)@Fe_3_O_4_ (A) survey spectra. (B) C 1s. (C) N 1s. (D) O 1s. (E) Zr 3d. (F) Fe 2p. (G) Zn 2p.

The elemental composition and chemical state of PCN-224(Zn)@Fe_3_O_4_ were analyzed using X-ray photoelectron spectroscopy (XPS). The XPS spectra of carbon (C), nitrogen (N), oxygen (O), iron (Fe), zinc (Zn), and zirconium (Zr) elements are presented in Figure 2. As shown in Figure 2B, the peaks at approximately 284.8, 286.4, and 288.8 eV in the binding energy are attributed to the C–C bond, C–O bond, and C=O bond, respectively. The peak at 398.5 eV in Figure 2C is identified as the C–N bond in PCN-224 (Zn), while the peak at 400.5 eV is attributed to the Zn–N bond within the porphyrin ring. The O 1s spectrum of PCN-224(Zn)@Fe_3_O_4_ (Figure 2D) can be fitted to three peaks at 530.1, 531.9, and 533.8 eV, which are assigned to Zr–O, C=O, and C–O groups, respectively.^43^^,^^44^ Meanwhile, the binding energy of the Fe–O bond is also around 530 eV. Figure 2E shows two distinct peaks at 182.9 eV and 185.3 eV, which are attributed to Zr 3d_5/2_ and Zr 3d_3/2_, respectively. Figure 2F shows that the Fe 2p fine structure spectrum fitting reveals six peaks, indicating the characteristic signals of Fe^2+^ and Fe^3+^. The peak at 709.65 eV corresponds to Fe^2+^ (2p_3/2_), the peak at 722.94 eV corresponds to Fe^2+^ (2p_1/2_), the peak at 710.92 eV corresponds to Fe^3+^ (2p_3/2_), and the peak at 724.53 eV corresponds to Fe^3+^ (2p_1/2_). The coexistence of these oxidation states is a characteristic of magnetite, distinguishing it from other iron oxides. Additionally, the presence of satellite contributions in the Fe^3+^ region supports the assignment of these peaks. The peak at 720.29 eV corresponds to the satellite peak of Fe^3+^ (2p_1/2_), and the peak at 732.32 eV corresponds to the satellite peak of Fe^3+^ (2p_3/2_). This confirms the presence of Fe_3_O_4_ and rules out the possibility of impurity phases such as FeO. Furthermore, the Zn 2p spectrum depicted in Figure 2G reveals two distinct binding energy peaks at approximately 1021.8 eV and 1044.9 eV, which can be attributed to Zn 2p_3/2_ and Zn 2p_1/2_, respectively.^45^

### Structural, morphological, and stability analyses of the nanofibers

**Figure 3:**
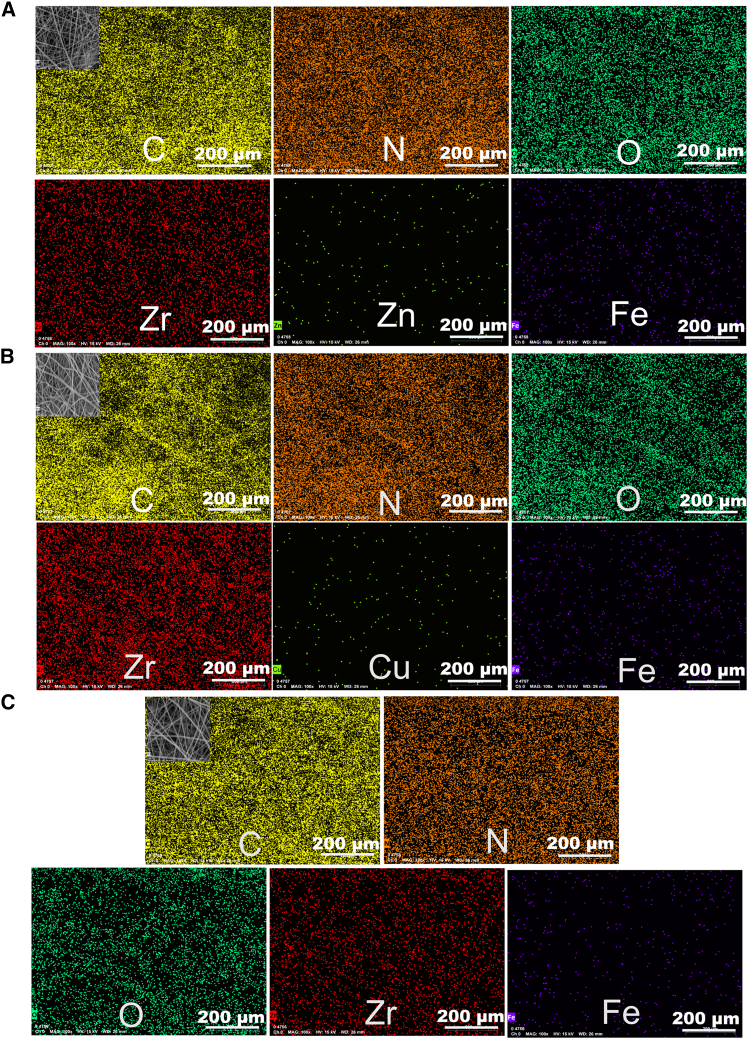
Element scanning distribution map (A) PCN-224(Zn)@Fe_3_O_4_/CS/PVA. (B) PCN-224(Cu)@Fe_3_O_4_/CS/PVA. (C) PCN-224(Fe)@Fe_3_O_4_/CS/PVA. All scale bars represent 200 μm.

Figures 3A–3C shows the elemental scan distribution of PCN-224(M)@Fe_3_O_4_/CS/PVA nanofibers. It can be observed that PCN-224(Zn)@Fe_3_O_4_/CS/PVA is composed of C, N, O, Zr, Zn, and Fe elements. The nanofiber of PCN-224(Cu)@Fe_3_O_4_/CS/PVA is composed of C, N, O, Zr, Cu, and Fe elements, while the nanofiber of PCN-224(Fe)@Fe_3_O_4_/CS/PVA is composed of C, N, O, Zr, and Fe elements. All elements of the three nanofibers are distributed evenly, indicating that PCN-224(M) and Fe_3_O_4_ are uniformly distributed in the fiber material.

**Figure 4:**
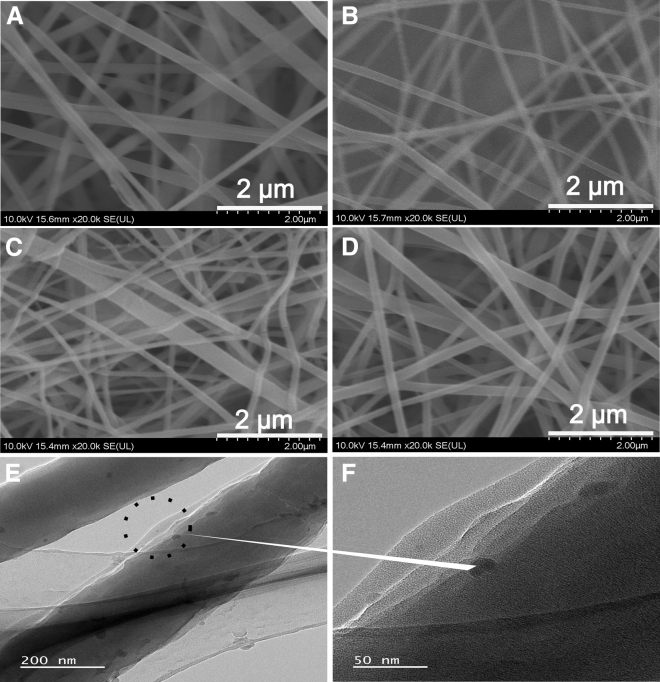
Morphology of nanofibers (A) SEM image of CS/PVA. (B) SEM image of PCN-224(Zn)@Fe_3_O_4_/CS/PVA. (C) SEM image of PCN-224(Cu)@Fe_3_O_4_/CS/PVA. (D) SEM image ofPCN-224(Fe)@Fe_3_O_4_/CS/PVA. (E and F) TEM images of PCN-224(Zn)@Fe_3_O_4_/CS/PVA. Scale bars: 2 μm (A, B, C, and D), 200 nm (E), and 50 nm (F).

The morphology of the prepared fibers was analyzed by SEM and transmission electron microscopy (TEM), and the results are shown in Figure 4. From the SEM image, it can be observed that the CS/PVA fiber’s surface is smooth, with good continuity and uniform thickness. After adding PCN-224(M)@Fe_3_O_4_, the fiber still maintains a good morphology, but the difference in fiber diameter increases. The surface of fiber becomes rough, and granular protrusions can be seen on the fiber surface. Comparing the SEM images of CS/PVA and PCN-224(M)@Fe_3_O_4_/CS/PVA, it can be seen that the addition of PCN-224(M)@Fe_3_O_4_ does not cause significant changes in the microstructure of the fiber. From the TEM images of PCN-224(Zn)@Fe_3_O_4_/CS/PVA electrospun fibers, it can be observed that the PCN-224(Zn)@Fe_3_O_4_ composite material is uniformly distributed within the fibers.

**Figure 5:**
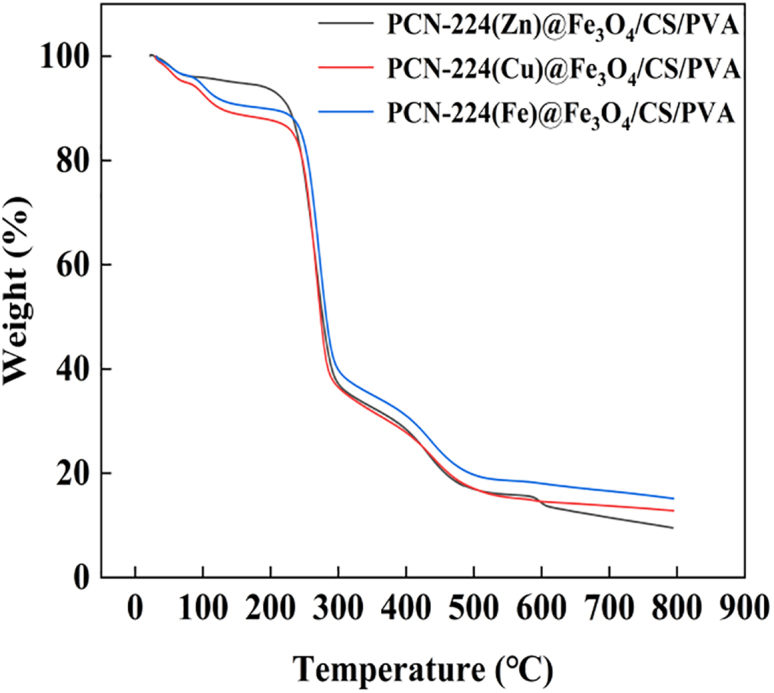
Thermogravimetric curves of PCN-224(M)@Fe_3_O_4_/CS/PVA

The thermogravimetric analysis of PCN-224(M)@Fe_3_O_4_/CS/PVA nanofibers is shown in Figure 5. Before 180°C, there was only a small amount of weight loss in the sample, which was caused by the evaporation of solvents and water in the sample due to heat. At this time, the fibers did not show significant decomposition; After heating to 180°C, due to the decomposition of PVA and CS in the fibers, the sample showed substantial weight loss. When the temperature exceeded 500°C, the weight loss rate was close to 80%, and the nanofibers were almost completely decomposed. It can be seen that all three nanofibers have good thermal stability.

### Exploration of antibacterial conditions

**Figure 6:**
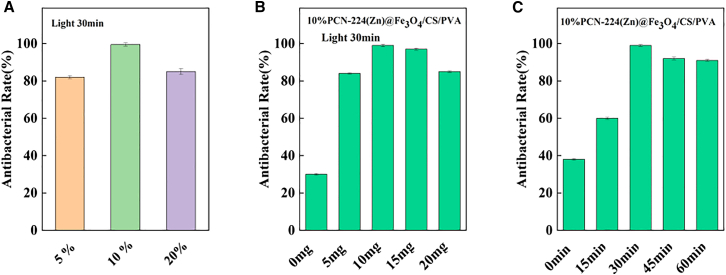
Antibacterial results of electrospun fibers against *E. coli* under different conditions (A) Effect of mass fraction of PCN-224(Zn)@Fe_3_O_4_. (B) Effect of catalyst dosage. (C) Effect of light irradiation time. Data are presented as mean ± SD, with three biological replicates per group.

Firstly, the effect of PCN-224(Zn)@Fe_3_O_4_ content in nanofibers on antibacterial efficacy was investigated. 10 mg of nanofibers loaded with different contents of PCN-224(Zn)@Fe_3_O_4_ (the mass fractions concentrations of PCN-224(Zn)@Fe_3_O_4_ in nanofibers were 5%, 10%, and 20%, respectively) were weighed and added to 10 mL of bacterial solution, and it was irradiated with a xenon lamp for 30 min. The antibacterial results are shown in Figure 6A. With an increase in the content of PCN-224(Zn)@Fe_3_O_4_, the antibacterial rate of nanofibers against *E. coli* first increased and then decreased. When the mass fraction of PCN-224(Zn)@Fe_3_O_4_ was 10%, the antibacterial rate was the highest (99.10%), and when the mass fraction was 20%, the antibacterial rate decreased. Secondly, the influence of the amount of catalyst on the antibacterial effect was explored: 0, 5, 10, 15, and 20 mg PCN-224(Zn)@Fe_3_O_4_/CS/PVA nanofibers were added to 10 mL of *E. coli* solution, and the light irradiation time was 30 min. The experimental results are shown in Figure 6B. With an increase in the amount of fiber added, the antibacterial effect of the material first increases and then decreases. When the mass of nanofiber added is 10 mg, the antibacterial effect is the best, and then the antibacterial rate decreases slightly. The reason why the antibacterial effect of nanofiber antibacterial agent decreases in the case of high content and large dosage could be that the magnetism of Fe_3_O_4_ in high concentration PCN-224(Zn)@Fe_3_O_4_ and the surface energy of PCN-224(M) promote the agglomeration of particles and reduce the effective active sites of the material.^46^ In addition, excessive catalyst will produce high local concentrations of ROS (such as OH and O^2−^), but ROS have a very short life, and they may react with each other to inactivate and also cause the decline of antibacterial effect.^47^ Finally, the effect of light irradiation time on the antibacterial effect of *E. coli* was explored, as shown in Figure 6C. The antibacterial rate of PCN-224(Zn)@Fe_3_O_4_/CS/PVA nanofibers against *E. coli* increased rapidly with the extension of light time from 0 to 30 min, and reached the peak at 30 min. This is mainly due to the continuous production of a large number of ROS by photosensitizer PCN-224(Zn) under light, which effectively destroys the bacterial structure. However, when the light irradiation time exceeded 30 min, the antibacterial rate decreased slightly. It could be that under the limited space and the number of bacteria, excessive ROS may react with each other and quench, resulting in the decline of antibacterial rate. According to the research results of antibacterial conditions, PCN-224(M)@Fe_3_O_4_/CS/PVA nanofibers with a mass of 10 mg and a mass fraction of 10% (PCN-224(M)@Fe_3_O_4_) were selected for follow-up experiments, and the light irradiation time was 30 min. The antibacterial effect of PCN-224(M)@Fe_3_O_4_/CS/PVA nanofibers on *E. coli*, *S. aureus*, and MRSA was explored.

### *In vitro* antimicrobial test

**Figure 7:**
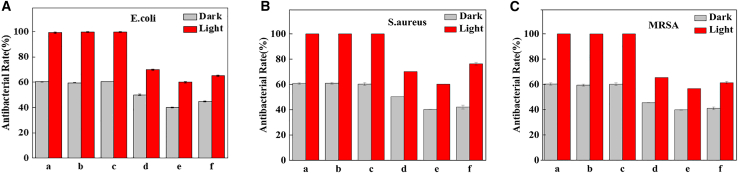
Antibacterial efficacy against various bacteria under light and dark conditions (A) *E. coli*. (B) *S. aureus*. (C) MRSA. Data are presented as mean ± SD, with three biological replicates per group. a, PCN-224(Zn)@Fe_3_O_4/_CS/PVA; b, PCN-224(Cu)@Fe_3_O_4_/CS/PVA; c, PCN-224(Fe)@Fe_3_O_4_/CS/PVA; d, PCN-224/PVA; e, Fe_3_O_4_/PVA; and f, CS/PVA.

**Figure 8:**
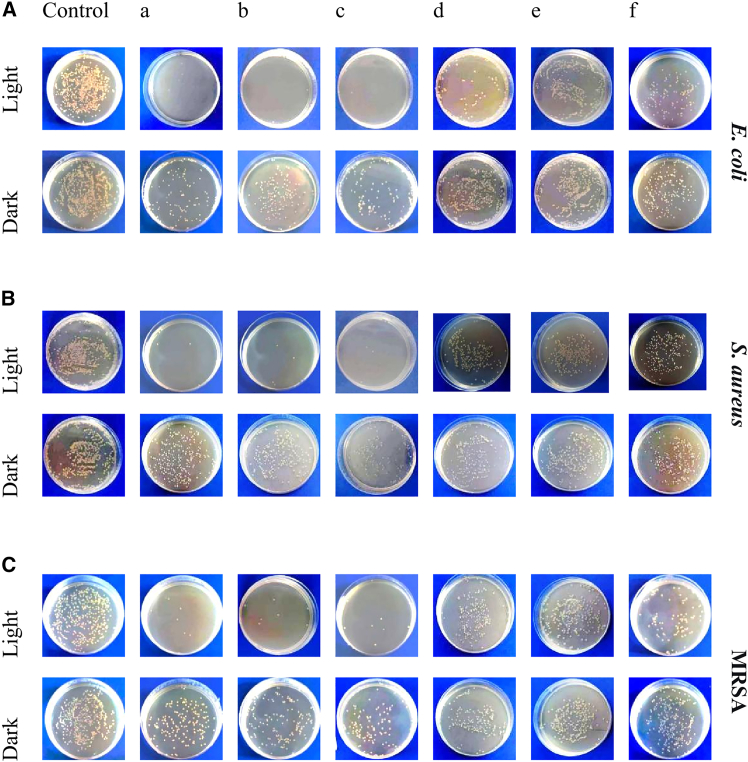
Photographs of the bacterial colonies under light and dark conditions (A) *E. coli*. (B) *S. aureus*. (C) MRSA. a, PCN-224(Zn)@Fe_3_O_4/_CS/PVA; b, PCN-224(Cu)@Fe_3_O_4_/CS/PVA; c, PCN-224(Fe)@Fe_3_O_4_/CS/PVA; d, PCN-224/PVA; e, Fe_3_O_4_/PVA; and f, CS/PVA.

The antibacterial performance of PCN-224(M)@Fe_3_O_4_/CS/PVA nanofibers under dark and light conditions was investigated with Gram-negative *E. coli*, Gram-positive *S. aureus*, and MRSA as the research objects, and the results are shown in Figure 7. The bacterial colony pictures of *E. coli*, *S. aureus*, and MRSA incubated with different PCN-224(M)@Fe_3_O_4_/CS/PVA nanofibers are also shown in Figure 8.

The antibacterial performance of PCN-224(M)@Fe_3_O_4_/CS/PVA against *E. coli* is shown in Figures 7A and 8A, and the results show the following obsevations:

(1) The antibacterial rates of all samples under light condition are higher than those under dark condition. Under dark condition, the antibacterial rate of PCN-224(M)@Fe_3_O_4_/CS/PVA against *E. coli* is about 60%, in which the antibacterial rate of PCN-224(Zn)@Fe_3_O_4_/CS/PVA is 61.37%, PCN-224(Cu)@Fe_3_O_4_/CS/PVA is 60.86%, and PCN-224(Fe)@Fe_3_O_4_/CS/PVA is 62.12%; the antibacterial rates of PCN-224(M)@Fe_3_O_4_/CS/PVA against *E. coli* are more than 99% after 30 min of light irradiation; and the antibacterial rates of PCN-224(Zn)@Fe_3_O_4_/CS/PVA, PCN-224(Cu)@Fe_3_O_4_/CS/PVA, and PCN-224(Fe)@Fe_3_O_4_/CS/PVA are 99.27% (equivalent to −log_10_(C/C_0_) = 2.52), 99.72% (equivalent to −log_10_(C/C_0_) = 2.52), and 99.92% (equivalent to −log_10_(C/C_0_) = 2.52), respectively. The antibacterial rate of PCN-224(M)@Fe_3_O_4_/CS/PVA nanofibers under light conditions is nearly 40% higher than that under dark conditions, which is mainly due to the strong photodynamic antibacterial effect of PCN-224(M) and the photothermal effect of Fe_3_O_4_. Under visible light irradiation, the porphyrin ligand in PCN-224(M), as a highly efficient photosensitizer, absorbs photons and then transitions to the excited singlet state, and then to a more stable triplet state through inter system transition. Subsequently, the photosensitizer in the triplet state transfers energy to the surrounding oxygen molecules, thereby producing a large number of cytotoxic ROS, especially singlet oxygen species. The introduced PTAT mechanism provides a powerful and complementary antibacterial pathway. ROS produced by photothermal effect, especially singlet oxygen, can oxidize the key cell components of bacteria, including lipids, proteins and nucleic acids in the cell membrane, leading to irreversible damage and rapid cell death.
(2) Comparison of antibacterial rates of nanofibers after 30 min of light irradiation suggests PCN-224(M)@Fe_3_O_4_/CS/PVA > PCN-224(M)/PVA > Fe_3_O_4_/PVA > CS/PVA. The antibacterial rate of PCN-224(M) and Fe_3_O_4_ composite nanofibers is significantly higher than that of single component PCN-224(M) and Fe_3_O_4_. This is because PCN-224(M)@Fe_3_O_4_/CS/PVA has the high-efficiency photodynamic effect of PCN-224(M), the photothermal effect of Fe_3_O_4_, as well as the contact sterilization ability of CS itself. Several antibacterial materials can work together to improve the antibacterial ability.

The antibacterial performance of PCN-224(M)@Fe_3_O_4_/CS/PVA nanofiber against *S. aureus* under dark and light conditions is shown in Figures 7B and 8B. The antibacterial rate of nanofiber materials against *S. aureus* is about 60% under dark conditions, including PCN-224(Zn)@Fe_3_O_4_/CS/PVA antibacterial rate 60.77%, PCN-224(Cu)@Fe_3_O_4_/CS/PVA antibacterial rate 60.96%, and PCN-224(Fe)@Fe_3_O_4_/CS/PVA antibacterial rate 60.30%. The antibacterial rates of nanofiber materials against *S. aureus* are more than 99.99% (equivalent to −log_10_(C/C_0_) = 2.54) after 30 min of light irradiation, which is about 40% higher than that under dark condition. Comparison of antibacterial rates of nanofibers after 30 min of light irradiation suggests PCN-224(M)@Fe_3_O_4_/CS/PVA > PCN-224(M)/PVA > Fe_3_O_4_/PVA > CS/PVA. The results show that the antibacterial rate of CS/PVA fiber against *S. aureus* is 76.31%, which is about 10% higher than that against *E. coli* (66.38%). This is because CS is a natural cationic polysaccharide with inherent antibacterial activity and good biocompatibility. As a natural cationic polymer antibacterial agent, it can destroy the bacterial cell membrane through positive charge, while the cell wall structure of *S. aureus* (Gram-positive bacteria) is more easily destroyed by CS, while *E. coli* (Gram-negative bacteria) is less sensitive to CS due to its outer membrane protection.^48^

The antibacterial effect of PCN-224(M)@Fe_3_O_4_/CS/PVA nanofiber against MRSA under dark and light conditions is shown in Figures 7C and 8C. The antibacterial rate of nanofiber material against MRSA under dark conditions is about 60%, including 60.31% of PCN-224(Zn)@Fe_3_O_4_/CS/PVA, 59.39% of PCN-224(Cu)@Fe_3_O_4_/CS/PVA, and 60.05% of PCN-224(Fe)@Fe_3_O_4_/CS/PVA. The antibacterial rates of nanofibers against MRSA after 30 min of light irradiation are 99.94% (equivalent to −log_10_(C/C_0_) = 2.53), 99.93% (equivalent to −log_10_(C/C_0_) = 2.52), and 99.96% (equivalent to −log_10_(C/C_0_) = 2.52) for PCN-224(Zn)@Fe_3_O_4_/CS/PVA, PCN-224(Cu)@Fe_3_O_4_/CS/PVA, and PCN-224(Fe)@Fe_3_O_4_/CS/PVA), respectively. The antibacterial rate of PCN-224(M)@Fe_3_O_4_/CS/PVA nanofiber is about 40% higher than that under dark condition, indicating that PCN-224(M)@Fe_3_O_4_/CS/PVA nanofiber could completely eliminate MRSA under visible light irradiation. Antibacterial experiments showed that the prepared PCN-224(M)@Fe_3_O_4_/CS/PVA nanofiber exhibited excellent synergistic antibacterial activity and biocompatibility. It could achieve excellent synergistic antibacterial activity through photodynamic and photothermal mechanisms under visible light irradiation and could play the role of CS bactericide itself, effectively killing gram-negative *E. coli*, gram-positive *S. aureus*, and MRSA.

### Photodynamic and photothermal effect

**Figure 9:**
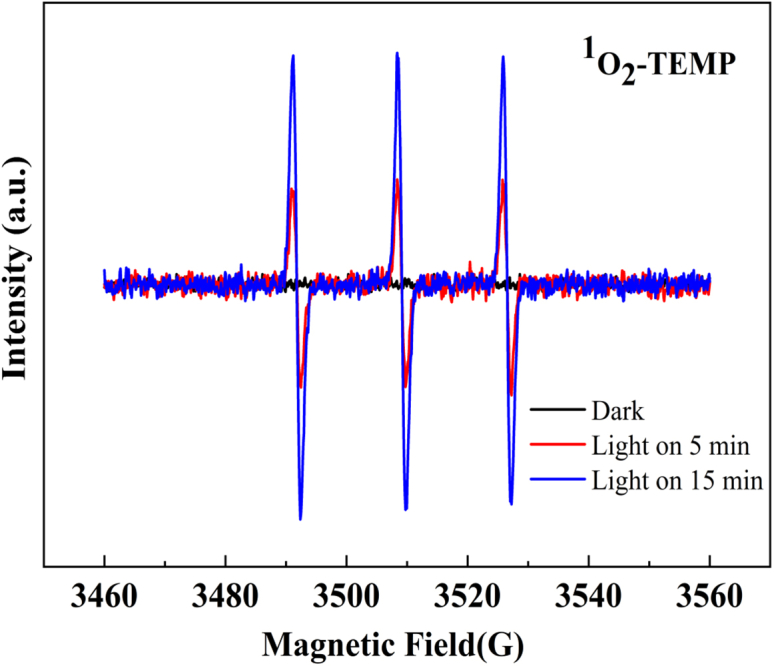
EPR spectra of PCN-224(Fe)@Fe_3_O_4_/CS/PVA for testing of ^1^O_2_

In our previous study, we found that the PMOFs could contributed to the transformation of ground-state oxygen (^3^O_2_) to ^1^O_2_.^49^ Herein, the production of ^1^O_2_ in PCN-224(Zn)@Fe_3_O_4_/CS/PVA was confirmed by using electron paramagnetic resonance (EPR) with 2,2,6,6-tetramethyl-4-piperidinol (TEMP) as a spin probe, and a typical 1:1:1 triplet peak for 4-hydroxy-2,2,6,6-tetramethylpiperidine 1-oxyl (TEMPOL) was observed upon light illumination (Figure 9). Moreover, the amount of ^1^O_2_ production significantly increased as the irradiation time was increased from 5 to 15 min. The stronger triplet peaks in the EPR spectrum for PCN-224(Zn)@Fe_3_O_4_/CS/PVA verified the higher yield of photoinduced ^1^O_2_.

**Figure 10:**
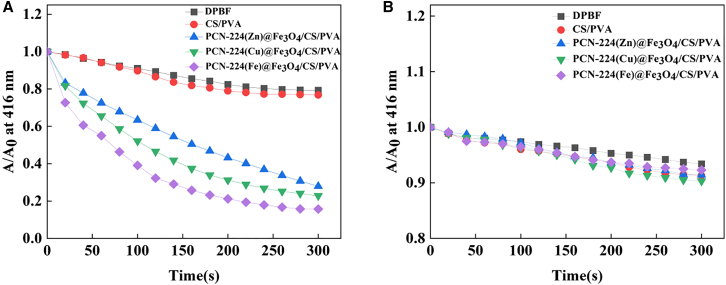
The absorbance of samples and DPBF (A) Under light irradiation. (B) Under dark condition.

To evaluate the ability of PCN-224(M)@Fe_3_O_4_/CS/PVA nanofibers to produce ROS under light irradiation, we used 1,3-diphenylisobenzofuran (DPBF) as a probe to quantitatively detect the ability of nanofibers to produce ^1^O_2_ under light and dark conditions. The results are shown in Figure 10. Under light irradiation, the absorbance of DPBF in the three kinds of PCN-224(M)@Fe_3_O_4_/CS/PVA nanofibers showed a continuous downward trend. After 300 s, the absorbance value decreased significantly. The absorbance value of DPBF in PCN-224(Fe)@Fe_3_O_4_/CS/PVA nanofibers decreased by 84.3%, that in PCN-224(Cu)@Fe_3_O_4_/CS/PVA nanofibers decreased by 77.2%, and that in PCN-224(Zn)@Fe_3_O_4_/CS/PVA nanofibers decreased by 77.2%. The absorbance of DPBF in CS/PVA nanofibers almost did not decrease. However, the absorbance of three kinds of PCN-224(M)@Fe_3_O_4_/CS/PVA nanofibers and CS/PVA decreased only slightly under dark conditions, which clearly showed that only under light conditions can PCN-224(M)@Fe_3_O_4_/CS/PVA nanofibers effectively produce ^1^O_2_. The results show that the ability for ^1^O_2_ generation is similar to the photodynamic antibacterial performance of the PCN-224(M)@Fe_3_O_4_/CS/PVA nanofibers.

**Figure 11:**
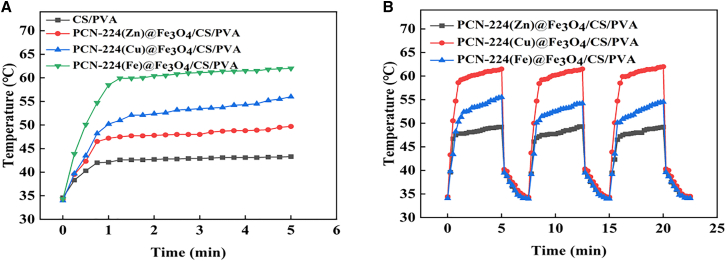
The photothermal experiment of nanofibers (A) Temperature change curves of CS/PVA, PCN-224(M)@Fe_3_O_4/_CS/PVA under light irradiation. (B) Photothermal stability curves of PCN-224(M)@Fe_3_O_4/_CS/PVA under light irradiation for three cycles.

PCN-224(M)@Fe_3_O_4_ shows strong and wide absorption characteristics in the 400–700 nm band, which lays the foundation for its photothermal application. As shown in Figure 11A, the temperature of PCN-224(M)@Fe_3_O_4_/CS/PVA nanofibers increased rapidly and then tended to plateau after 5 min of light irradiation. Among them, the maximum temperature of PCN-224(Zn)@Fe_3_O_4_/CS/PVA was 49.7°C, that of PCN-224(Cu)@Fe_3_O_4_/CS/PVA was 56.0°C, that of PCN-224(Fe)@Fe_3_O_4_/CS/PVA was 62.0°C, while that of CS/PVA was only 43.3°C. This indicates that PCN-224(M)@Fe_3_O_4_/CS/PVA nanofibers have potential application in photothermal conversion. As shown in Figure 11B, after three photothermal cycles, the maximum temperature corresponding to the nanofiber material has no noticeable change, indicating that the nanofiber material has good photothermal stability.^50^ The experimental results indicate that under light irradiation, PCN-224(M)@Fe_3_O_4_/CS/PVA nanofibers can rapidly increase the temperature to 50°C–60°C. The photothermal effect significantly enhances the catalytic production of ROS by the nanofibers, causing lethal damage to bacteria. Combined with the excellent photodynamic effect of PCN-224(M), the growth of bacteria can be completely inhibited.

### Biological safety

**Figure 12:**
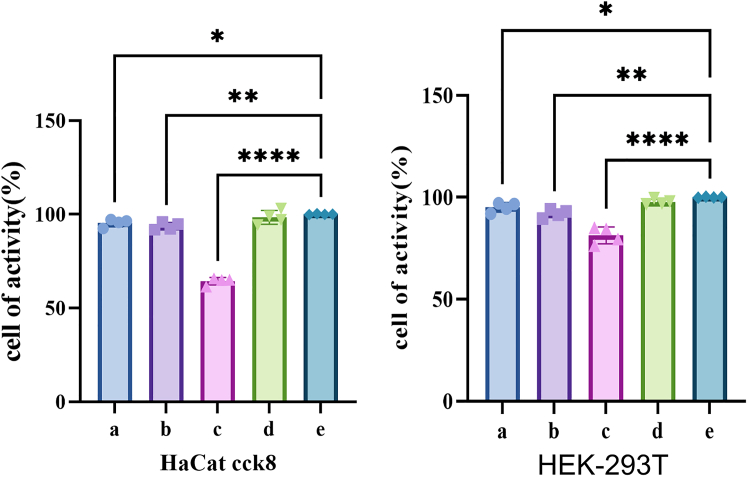
The biological safety experiment of nanofibers The biological safety experiments on HEK-293T (A) and HaCat (B) cells. (a, PCN-224(Zn)@Fe_3_O_4_/CS/PVA; b, PCN-224(Cu)@Fe_3_O_4_/CS/PVA; c, PCN-224(Fe)@Fe_3_O_4_/CS/PVA; d, CS/PVA; and e, ck). Cell viability of HaCat and HEK-293T cells after incubation with composite fiber extracts was measured via the CCK-8 assay. Data are expressed as mean ± SD. Statistical analyses were carried out by one-way ANOVA with Tukey’s multiple comparisons test. ∗*p* < 0.05, ∗∗*p* < 0.01, ∗∗∗∗*p* < 0.0001.

The cytotoxicity of nanofibers was determined by the CCK-8 method. The biological safety of nanofibers was evaluated by studying the cell activity of PCN-224(M)@Fe_3_O_4_/CS/PVA nanofibers co-cultured with HEK-293T and HaCat cells. The results are shown in Figure 12. PCN-224(Zn)@Fe_3_O_4_/CS/PVA and PCN-224(Cu)@Fe_3_O_4_/CS/PVA nanofibers had little effect on the activity of HEK-293T cells, and the cell survival rates were 95.01% and 92.04%, respectively. PCN-224(Fe)@Fe_3_O_4_/CS/PVA had a slightly greater effect on the activity of HEK-293T cells, and the survival rate of HEK-293T cells was 81.30%. PCN-224(Zn)@Fe_3_O_4_/CS/PVA and PCN-224(Cu)@Fe_3_O_4_/CS/PVA nanofibers also showed good biological safety on HaCat cells, and the cell survival rates were 95.40% and 93.70%, respectively. In contrast, PCN-224(Fe)@Fe_3_O_4_/CS/PVA exerted certain phototoxicity, and the survival rate of HaCat cells was 64.32%. From the biological safety study of PCN-224(M)@Fe_3_O_4_ nanofibers on HEK-293T and HaCat cells, it can be seen that PCN-224(Fe)@Fe_3_O_4_/CS/PVA in this series of composite fibers exerts certain phototoxicity. From the photodynamic and photothermal experiments, it can also be seen that PCN-224(Fe)@Fe_3_O_4_/CS/PVA can produce more ROS and higher temperature under light. Hence, the phototoxicity is also higher than that of PCN-224(Zn)@Fe_3_O_4_/CS/PVA and PCN-224(Cu)@Fe_3_O_4_/CS/PVA. In general, this series of nanofiber materials has no apparent cytotoxic effect and good biological safety, which has a particular application prospect in the biomedical field. The antibacterial materials prepared in this study encompass both inorganic antibacterial agents, such as Fe_3_O_4_ and PCN-224(M) as well as organic materials CS and PVA. The inorganic antibacterial agents Fe_3_O_4_ and PCN-224(M) exhibit stable properties and do not decompose or produce toxic by-products under irradiation. Furthermore, the content of PCN-224(M)@Fe_3_O_4_ is only 10%, and *in vitro* experiments have demonstrated its good biological safety. The polymer materials CS and PVA serve as substrates with good radiation tolerance, which can reduce the direct damage of radiation to the antibacterial agent and prevent the degradation of the matrix caused by radiation, thereby preventing the production of harmful substances.

### Stability and reusability

**Figure 13:**
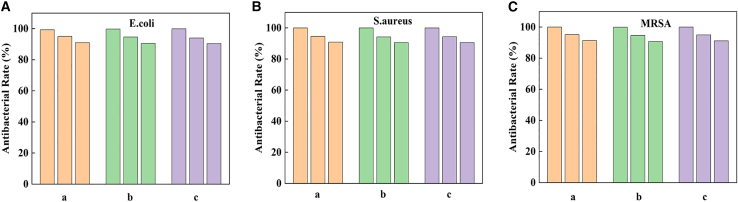
Antibacterial effect of PCN-224(M)@Fe_3_O_4/_CS/PVA for three cycles (A) *E. coli*. (B) *S. aureus*. (C) MRSA. a, PCN-224(Zn)@Fe_3_O_4_/CS/PVA; b, PCN-224(Cu)@Fe_3_O_4_/CS/PVA; and c, PCN-224(Fe)@Fe_3_O_4_/CS/PVA.

The stability and recyclability of photocatalysts are critical for practical applications. Therefore, recycling experiments were conducted by recovering PCN-224(M)@Fe_3_O_4_/CS/PVA after its engagement in the antibacterial experiments. The antibacterial results are shown in Figure 13. After three cycles of experiments, the antibacterial rates of the three PCN-224(M)@Fe_3_O_4_/CS/PVA nanofibers against *E. coli*, *S. aureus*, and MRSA were more than 90%, indicating that PCN-224(M)@Fe_3_O_4_/CS/PVA nanofibers are not only efficient and broad-spectrum antibacterial materials but also environment-friendly and sustainable antibacterial solution with great potential for practical application because of excellent cycle stability and convenient recovery characteristics.

## Discussion

In this paper, a series of new photocatalytic antibacterial fibers, PCN-224(M)@Fe_3_O_4_/CS/PVA, has been prepared. Through its exceptional ability to produce ROS, it has achieved efficient bacterial inactivation. Compared with PCN-224(M)/PVA, Fe_3_O_4_/PVA and CS/PVA fibers, PCN-224(M)@Fe_3_O_4_/CS/PVA nanofibers showed significantly improved photocatalytic antibacterial activity against *S. aureus*, *E. coli*, and MRSA, and the sterilization rate was close to 100%. The mechanism study shows that the nanofibers can produce reactive oxygen under light irradiation and have stable photothermal properties. The introduction of CS effectively improved the contact antibacterial effect and biocompatibility of the material, and the fibrous material had no apparent cytotoxicity and showed good biological safety.

PCN-224(M)@Fe_3_O_4_/CS/PVA not only has high-efficiency and broad-spectrum antibacterial properties but also solves the key problem of difficult recovery of traditional antibacterial materials in the integration of photodynamic antibacterial, photothermal antibacterial, and contact sterilization. The material has broad application prospects in medical dressings, water treatment, and other fields. Future research will focus on optimizing the preparation process of the material, further improving its antibacterial properties, reducing costs, and promoting its practical application.

### Limitations of the study

In this study, we prepared a series of PCN-224(M)@Fe_3_O_4_/CS/PVA electrospun fibers, which exhibit high antibacterial efficiency against *S. aureus*, *E. coli*, and MRSA. These fibers have potential applications in antibacterial dressings, food packaging, and other fields. The material contains multiple components, including PCN-224(M) and Fe_3_O_4_, both of which can generate ROS under light irradiation and exhibit a certain photothermal effect. CS, on the other hand, is a contact antibacterial material. Therefore, the antibacterial mechanism of the material is relatively complex. Further research on the antibacterial mechanism can provide a theoretical basis for enhancing the antibacterial performance of the material. Additionally, due to limited conditions, this study did not conduct animal experiments and *in vivo* safety assessments, which restricts the practical application of the material.

## Resource availability

### Lead contact

Further information and requests for resources and regents should be directed to and will be fulfilled by the lead contact, Erjun Sun (sunerjun@ccsfu.edu.cn).

### Materials availability

Materials used in the study are commercially available.

### Data and code availability

• All data reported in this article will be shared by the lead contact upon reasonable request.
• This article does not report original code.
• Any additional information required to reanalyze the data reported in this article is available from the lead contact upon reasonable request.

## Acknowledgments

This research was supported by 10.13039/100007847Natural Science Foundation of Jilin Province (grant no. YDZJ202601ZYTS236). The authors would like to thank Dr. Taotao Mi of Tongji University for biological safety testing and SCI-GO (www.sci-go.com) for the XPS, SEM, TEM, and EPR analyses.

## Author contributions

Conceptualization, E.S.; methodology, C.H., Q.L., and L.W.; investigation, F.N.; writing – original draft, C.H.; writing – review and editing, E.S and C.H.; funding acquisition, E.S.; resources, E.S and Q.L; supervision, E. S. All authors have read and agreed to the published version of the article.

## Declaration of interests

The authors declare no competing interests.

## STAR★Methods

### Key resources table

**Table:** 

REAGENT or RESOURCE	SOURCE	IDENTIFIER
**Bacterial and virus strains**		

***E coli*** (CMCC44103)	Beina Biological Collection Center	N/A
***S. aureus*** (CMCC26003)	Beina Biological Collection Center	N/A
**MRSA** (ATCC43300)	Beina Biological Collection Center	N/A

**Chemicals, peptides, and recombinant proteins**		

1,3-diphenylisobenzofuran (DPBF)	Shanghai Aladdin Biochemical Technology Co., Ltd.	CAS:5471-63-6
N. N-dimethylformamide (DMF)	Shanghai Aladdin Biochemical Technology Co., Ltd.	CAS:68-12-2
anhydrous ethanol (99.7%)	Shanghai Chemical Reagent Co., Ltd.	CAS:64-17-5
anhydrous methanol (99.8%)	Shanghai Chemical Reagent Co., Ltd.	CAS:67-56-1
acetone	Shanghai Chemical Reagent Co., Ltd.	CAS:67-64-1
ZrCl_4_	Shanghai Chemical Reagent Co., Ltd.	CAS:10026-11-6
Benzoic acid	Shanghai Chemical Reagent Co., Ltd.	CAS:65-85-0
Fe_3_O_4_ NP_S_	Shanghai Aladdin Biochemical Technology Co., Ltd.	CAS:1317-61-9, ≥99% metals basis, powder, 20 nm

**Experimental models: Cell lines**		

HaCaT (Immortalized human keratinocytes)	Cell Center, Institute of Basic Medical Sciences, Chinese Academy of Sciences, China	N/A
HEK-293T (human embryonic kidney 293 cell)	Cell Center, Institute of Basic Medical Sciences, Chinese Academy of Sciences, China	N/A

### Method details

#### Materials

All of the chemicals were obtained from commercial sources and used without further purification, except tetracarboxyphenyl porphyrin metal complexes (Zn-TCPP, Cu-TCPP, Fe-TCPP) were synthesized according to the literature in our laboratory.^51^ Nutrient agar (NA), nutrient broth (NB), tryptone soy agar (TSA), and tryptone soy broth (TSB) were purchased from Qingdao Haibo Biotechnology Co., Ltd.

#### Preparation of PCN-224(M) and PCN-224(M)@Fe_3_O_4_ composite materials

The synthesis procedure for PCN-224(M) was performed following the methods described in ref.^52^ Initially, 50 mg of MTPPP, 78 mg of ZrCl_4_ and 2700 mg of BA were dispersed in 16 mL of DMF and subjected to sonication until complete dissolution. Subsequently, this mixture was transferred to a Teflon reactor and heated at 120°C for 48 hours. After cooling to room temperature, the purple cubic crystals were collected by centrifugation and washed several times with DMF and acetone, respectively. The collected product was placed in acetone for activation treatment and dried at 80°C for 10 hours. PCN-224(M) (M = Zn, Cu, Fe) was obtained after drying.

The synthesis method of PCN-224(M)@Fe_3_O_4_ composite material is similar to that of PCN-224(M). Initially, 50 mg of Fe_3_O_4_ was dispersed in 16 mL of DMF and sonicated for 30 minutes to disperse evenly. Then, 50 mg of MTPCP, 78 mg of ZrCl_4_, and 2700 mg of BA were added to this solution sequentially and sonicated until the mixture turned into a homogeneous solution. Subsequently, the solution was transferred to a Teflon reactor and heated at 120°C for 48 hours. After cooling to room temperature, the deep purple cubic crystals were collected by centrifugation, washed several times with DMF and acetone, and the collected product was placed in acetone for activation treatment before being dried in an 80°C oven for 10 hours. After drying, PCN-224(M)@Fe_3_O_4_ composite materials were obtained, which were: PCN-224(Zn)@Fe_3_O_4_, PCN-224(Cu)@Fe_3_O_4_, and PCN-224(Fe)@Fe_3_O_4_.

#### Preparation of PCN-224(M)@Fe_3_O_4_/CS/PVA nanofibers

0.6 g of PVA and 9.4 g of H_2_O were added into a glass vial, then stirred at 90°C to obtain a homogeneous solution A. 0.3 g CS, 7.76 g acetic acid, and 1.94 g H_2_O were added into another glass vial, stirred and dissolved at room temperature to obtain solution B. 2.1 g of solution B was added dropwise to 4.9 g of solution A while stirring, then a certain mass of PCN-224(M)@Fe_3_O_4_ was added and continue stirred overnight to obtain a homogeneous viscous spinning solution. PCN-224(M)@Fe_3_O_4_/CS/PVA nanofibers were prepared using electrospinning technology. PCN-224(M)/PVA, Fe_3_O_4_/PVA, and CS/PVA nanofibers were prepared using the same method.

#### Characterizations

FT-IR spectra were collected from KBr pellets on an IS 50 spectrophotometer. UV-vis diffuse reflectance spectra (DRS) were collected using a Cary 300 UV-vis spectrophotometer equipped with an integrated sphere, and BaSO_4_ was used as a reference for the measurements. Thermogravimetric analysis (TGA) was carried out under N_2_ atmosphere on a TG-7 instrument at a heating rate of 10 °C min^−1^. Powder X-ray diffraction (PXRD) data were recorded on a Bruker D2 phaser. Scanning electron microscopy (SEM) images were recorded on a HITACHI SU8100 equipment equipped with an energy dispersive X-ray (EDX) detector, and the samples were deposited onto ultrathin carbon films on copper grids. The morphologies of PCN-224(M)@Fe_3_O_4_/CS/PVA employing transmission electron microscope (TEM, JEM-F200 Ultim Max 80) were observed. X-ray photoelectron spectroscopy (XPS) was employed for structure characterization on a Thermo K-Alpha multifunctional imaging electron spectrometer. The HSX-F300 xenon lamp has a power of 300 W (0.3 W/cm^2^) and covers the full wavelength range from 300 nm to 2500 nm.

#### Antibacterial experiment

The PCN-224(M)@Fe_3_O_4_/CS/PVA nanofibers were cut into small discs of 1 × 1 cm, and the antibacterial activities of the nanofibers on *E coli, S. aureus and* MRSA were explored using the coating plate method. First, the frozen *E. coli* bacterial solution was dissolved in a 37°C water bath, then transferred to 100 mL of NB medium. The solution was shaken at 37°C for 12 hours. Then the bacterial solution was diluted to 10^6^ CFU/mL with NB liquid medium, and a certain mass of nanofibers was added to 10 mL of bacterial dilution solution. After repeated blowing and beating, the bacterial solution was transferred to dark conditions for incubation for 30 minutes. Afterwards, the xenon lamp was used to continuously illuminate for a certain period of time and let it stand for 30 minutes. Finally, the bacterial solution was subjected to a series of gradient dilutions. 100 μL of the diluted bacterial solution was evenly coated on the surface of the solid culture medium and incubated at 37°C for 12 hours. The bacterial survival rate was detected using the colony counting method and the parallel experiments were performed thrice on each sample. The antibacterial rate was calculated according to Equation 1.

(Equation 1) $$\text{Antibacterial}\;\text{Rate}=\left(N_{b}-N_{t}\right)/N_{b}\times 100\%$$

Among them, N_b_ refers to the number of bacterial colonies without samples, and N_t_ refers to the number of bacterial colonies with samples.

Using the same method, the antibacterial activity of PCN-224(M)@Fe_3_O_4_/CS/PVA nanofibers on *S. aureus* and MRSA was investigated.

### Quantification and statistical analysis

All quantitative data in this study were obtained from three independent experimental replicates (*n* = 3) and expressed as mean ± standard deviation (SD).

XPS spectra were analyzed via Advantage software, and biosafety data were processed with GraphPad Prism. Plotting and statistical analysis of the remaining datasets were performed using Origin software. No other advanced statistical approaches or analytical software were utilized in this work.

### Additional resources

This study has not generated or contributed to a new website/forum.
